# Screening of Additive Manufactured Scaffolds Designs for Triple Negative Breast Cancer 3D Cell Culture and Stem-Like Expansion

**DOI:** 10.3390/ijms19103148

**Published:** 2018-10-12

**Authors:** Emma Polonio-Alcalá, Marc Rabionet, Antonio J. Guerra, Marc Yeste, Joaquim Ciurana, Teresa Puig

**Affiliations:** 1 New Therapeutic Targets Laboratory (TargetsLab)—Oncology Unit, Department of Medical Sciences, Faculty of Medicine, University of Girona, Emili Grahit 77, 17003 Girona, Spain; emma.polonio@udg.edu (E.P.-A.); m.rabionet@udg.edu (M.R.); 2 Product, Process and Production Engineering Research Group (GREP), Department of Mechanical Engineering and Industrial Construction, University of Girona, Maria Aurèlia Capmany 61, 17003 Girona, Spain; antonio.guerra@udg.edu; 3 Biotechnology of Animal and Human Reproduction (TechnoSperm), Department of Biology, Institute of Food and Agricultural Technology, University of Girona, Pic de Peguera 15, 17003 Girona, Spain; marc.yeste@udg.edu

**Keywords:** 3D printing, three-dimensional cell culture, scaffolds, PLA, TNBC, breast cancer stem cells

## Abstract

Breast cancer stem cells (BCSCs) are tumor-initiating cells responsible for metastasis and tumor reappearance, but their research is limited by the impossibility to cultivate them in a monolayer culture. Scaffolds are three-dimensional (3D) cell culture systems which avoid problems related with culturing BCSC. However, a standardized scaffold for enhancing a BCSC population is still an open issue. The main aim of this study is to establish a suitable poly (lactic acid) (PLA) scaffold which will produce BCSC enrichment, thus allowing them to be studied. Different 3D printing parameters were analyzed using Taguchi experimental design methods. Several PLA scaffold architectures were manufactured using a Fused Filament Fabrication (FFF) 3D printer. They were then evaluated by cell proliferation assay and the configurations with the highest growth rates were subjected to BCSC quantification by ALDH activity. The design SS1 (0.2 mm layer height, 70% infill density, Zigzag infill pattern, 45° infill direction, and 100% flow) obtained the highest proliferation rate and was capable of enhancing a ALDH+ cell population compared to 2D cell culture. In conclusion, the data obtained endorse the PLA porous scaffold as useful for culturing breast cancer cells in a microenvironment similar to in vivo and increasing the numbers of BCSCs.

## 1. Introduction

Breast cancer is the most commonly diagnosed cancer and the second cause of cancer death among women [1]. Triple negative breast cancer (TNBC) is characterized by the lack of estrogen and progesterone receptors and no overexpression of human epidermal growth factor receptor-2 [2]. It accounts for 15–20% of the patients diagnosed with breast cancer [3]. TNBC is very aggressive and has a poor prognosis due to the young age of the patients, its high metastasis and relapse incidence and its higher mortality in comparison with the other breast cancer subtypes [4]. Cytotoxic chemotherapy is the main treatment against TNBC owing to the lack of a validated targeted therapy [5]. Cancer stem cells (CSCs) are a tumor-initiating subpopulation responsible for tumor recurrence as a result of their resistance to the anti-cancer therapy [6,7,8,9]. They share similar features with normal stem cells, such as the ability to self-renew and generate the bulk of the tumor [10]. CSCs have the capacity to grow in suspension where they form spheres and they also have an enhanced activity of the aldehyde dehydrogenase (ALDH) enzyme [11,12]. Hence, a breast cancer stem cell (BCSC) population could become a potential target for breast cancer treatment, and in particular, the TNBC subtype.

Traditionally, cells are cultivated on two-dimensional (2D) supports, however these supports modify the shape of the cell, and consequently, gene expression and protein regulation are altered compared with cells surrounded by a physiological environment [13,14]. In vivo, the extracellular matrix (ECM), which is a three-dimensional (3D) structure composed of fibrous proteins and molecules, surrounds cells and plays a key role in cell regulation [15]. For that reason, 3D cell culture systems such as scaffolds, have emerged as an alternative to mimicking in vivo cell conduct, thus making experiment results more reliable [16]. Different methodologies exist for manufacturing these structures. For instance, fused filament fabrication (FFF) is an additive manufacturing (AM) technique where a 3D printer melts and deposits the material in successive layers [17]. The porous scaffolds obtained allow seeded cells to adopt their natural morphology and interact with their adjacent cells in a 3D tissue-like environment [16]. While a wide range of materials has been employed, it is biopolymers that are extensively used for 3D cell culture [16,18]. Poly (lactic acid) (PLA) is an ideal biopolymer for biomedical and cell culture applications thanks to its biocompatibility, safe degradation products, high strength, and good blend-compatibility with others polymers [19,20,21], and as such, has been used for porous scaffolds [21]. 

While there is considerable interest in BCSC population, its study is limited because 2D culture induces their differentiation during cell propagation, losing their stem properties [22,23], as well as their low representation within the tumor [24,25]. The environment may also influence the CSC differentiation state [22,26]. 3D culture systems, like scaffolds, offer a physical structure that mimics the in vivo environment and overcomes the problems related to BCSC culture. Moreover, researchers have demonstrated that scaffolds produce an enrichment of this malignant subpopulation, thus facilitating its investigation [23,27,28].

Despite the research that has already been carried out in this field, many concerns remain and manufacturing a 3D scaffold with the appropriate properties to achieve optimal BCSC enrichment is still an open issue. As the printing procedure involves many input parameters, this complicates the settings of all the variables. Therefore, this current work aims to analyze the most important parameters through a robust design of experiment method known as the Taguchi method. Different 3D printing parameters-layer height, infill density, infill pattern, infill direction, and material flow-were studied. Several PLA scaffold designs were fabricated with a FFF 3D printer and then evaluated through a cell proliferation assay using MDA-MB-231 triple negative breast carcinoma cells. The architectures with the highest growth rates were subjected to BCSC quantification. The data obtained supports the idea that PLA porous scaffold are useful for culturing and proliferating breast cancer cells in a microenvironment that preserves stemness and increase the BCSC subpopulation in a short timeframe.

## 2. Results

### 2.1. PLA Scaffolds Production and Characterization

To accommodate an optimal three-dimensional cell culture, the main aim was to develop a scaffold architecture which affords a high breast cancer cell proliferation rate. For this purpose, several values of the selected parameters (layer height, infill density, infill pattern, infill direction, and flow) were tested to find the optimal ones. Using the Taguchi experimental design method, twenty-seven scaffold configurations were manufactured and then analyzed (Table 1). To perform the characterization and cell proliferation assays, at least ten copies of each configuration were printed. 

**Figure 1:**
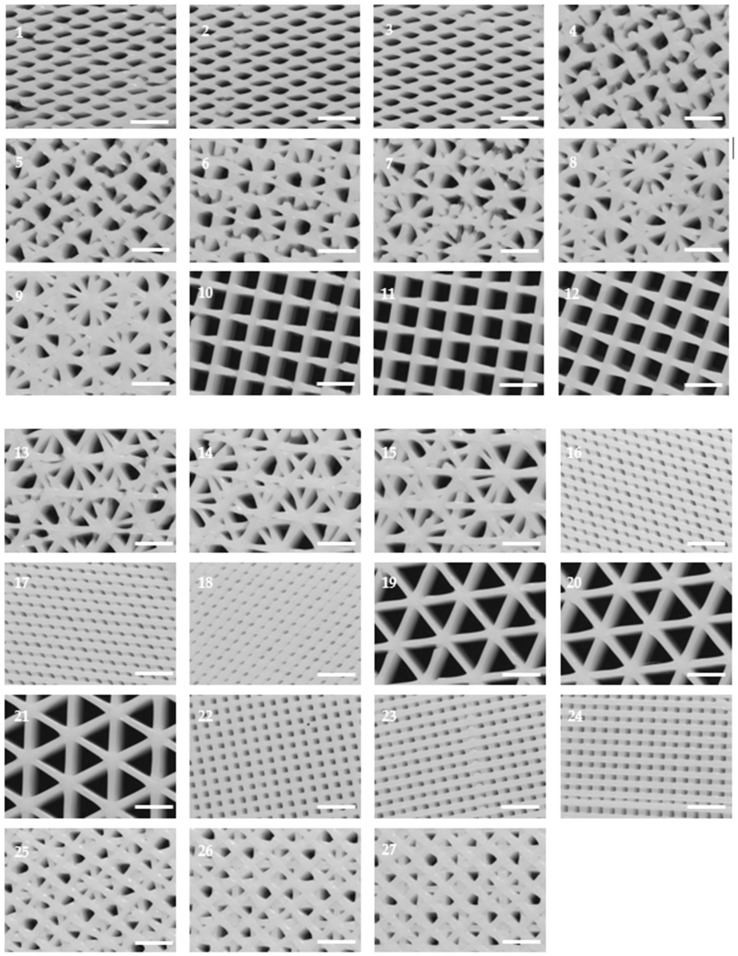
Microscopic characterization of PLA scaffold configurations. Top side was visualized under an optical microscope and images were used to calculate pore area and filament diameter. (Scale bar: 2 mm).

As shown in Figure 1, different scaffold typologies were manufactured. Some of them had a regular pattern and their pores had similar areas, for example, designs 10 or 22, while others, for example, configurations 6 or 14, had a non-regular pattern with pores of different areas. 

To study the microscopic architecture of the scaffolds produced, only the top side was analyzed by optical microscopy because the first printed layer (on the bottom) was different from the rest of layers. Generally, the filament diameter was a little bigger and irregular because of flattening.

The results observed for PLA scaffold characterization (Table 2) were as expected. In general, the filament diameter was approximately 0.3 mm in all designs because of the 0.3 mm nozzle chosen and the very similar flow rate (80–100%). Also, it was observed when the quantity of printed material increased, the pore area decreased, while the filament diameter was enlarged.

Moreover, small pore areas were reached, with average values between 0.040 and 0.402 mm^2^. However, some configurations showed bigger pore areas greater than 1 mm^2^. Concrete examples of this are architectures 10, 11, and 12 (50% infill density, grid pattern, and 90° direction) and 19, 20, and 21 (50% infill density, triangles pattern, and 60° direction).

### 2.2. Cell Proliferation Assay

#### 2.2.1. Selecting the Optimal Values for Each Parameter Tested

**Figure 2:**
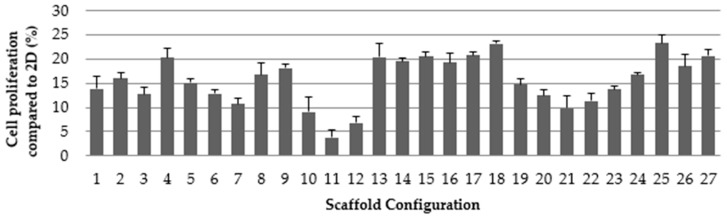
MDA-MB-231 cell proliferation for each PLA scaffold configuration (3D) as compared to a two-dimensional surface (2D) (*n* = 3).

Once all the configurations had been printed, a cell proliferation assay was performed for all the architectures to select the scaffolds that provide optimal cell growth rates, as depicted in Figure 2. The cell proliferation that was obtained in 3D cell culture was then normalized to the growth rate exhibited for 2D cell culture. This characterization was required to evaluate whether scaffolds could be used for BCSC experiments or not, as having enough cells attached with which to perform these experiments is a mandatory requirement. Hence, MDA-MB-231 cells were cultured for three days on 2D adherent surfaces and on PLA scaffolds. Results were then analyzed using Quantum XL software.

As seen in Figure 2, architectures with larger pore areas obtained the lowest cell proliferation rates (for instance, 10, 11, and 12), whereas design 18, which had the smallest average pore area, presented the highest proliferation. Scaffolds with irregular pore areas also had good cell proliferation rates, for example, architectures 4 and 25.

**Figure 3:**
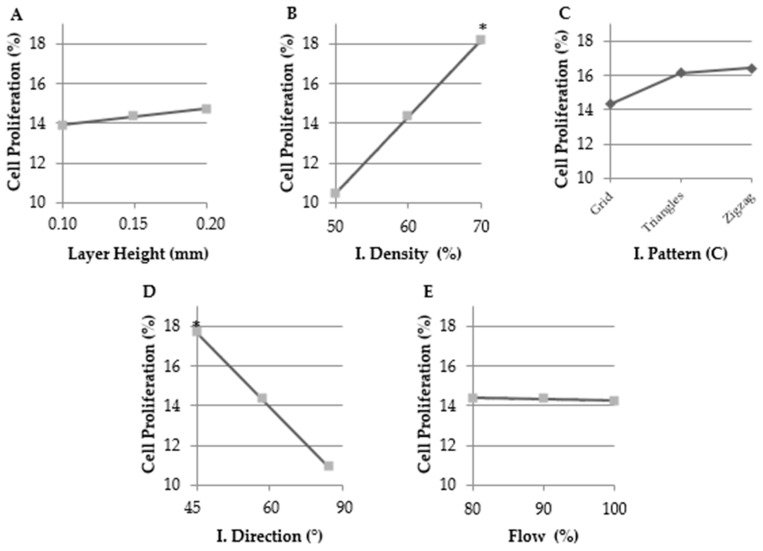
Main effect plots for each parameter on cell proliferation rate obtained through Quantum XL software. (**A**) Layer height. Samples printed with 60% infill density, grid infill pattern, 60° infill direction and 90% flow. (**B**) Infill density. Samples printed with 0.15 mm layer height, grid infill pattern, 60° infill direction, and 90% flow. The value of 70% significantly increased cell proliferation. (**C**) Infill pattern. Samples printed with 0.15 mm layer height, 60% infill density, 60° infill direction, and 90% flow. Zigzag pattern showed a light trend to obtain a higher proliferation rate. (**D**) Infill direction. Samples printed with 0.15 mm layer height, 60% infill density, grid infill pattern, and 90% flow. The value of 45° significantly increased cell proliferation. (**E**) Flow. Samples printed with 0.15 mm layer height, 60% infill density, grid infill pattern, and 60° infill direction. Significant differences are indicated as * (*p* < 0.05).

In Figure 3, the results obtained indicated that infill density and infill direction parameters had a significant influence on cell proliferation with the optimal tested values being 70% (designs 7–9, 16–18, and 25–27) and 45° (designs 1–3, 13–15, and 25–27), respectively. 

Therefore, three more scaffold configurations, called SS (selected scaffold), were designed and synthesized with the previously determined optimal value parameters but differing in pattern (see Table 3). The main aim of these new structures was to develop a scaffold with optimal value parameters that would reach a maximum cell growth rate. The value selected for layer height was 0.2 mm thanks to its positive trend. Despite its negative tendency, the chosen flow parameter value was 100% because printing at 100% flow presented fewer difficulties than at 80%, which caused some issues like nozzle obstruction, etc. However, the SS3 scaffold design was not printed because the selected values were the same as those for configuration 27, which had already been tested.

#### 2.2.2. Selected Value Verification and Final Selection of the Optimal Designs

At least ten copies of the new scaffold configurations SS1 and SS2 were printed and a cell proliferation assay was also performed for MDA-MB-231 cells following the same methodology as that used for the other 27 architectures. 

**Figure 4:**
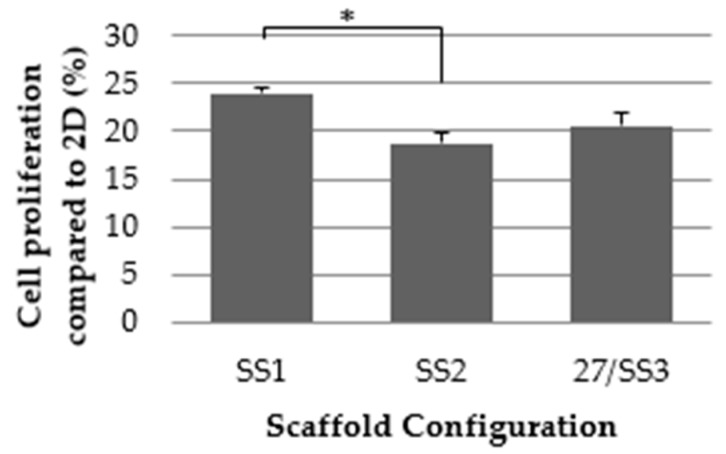
MDA-MB-231 cell proliferation assay of PLA scaffold configurations SS1, SS2 and 27/SS3 (3D) compared to two-dimensional surface (2D) (*n* = 3). Significant differences are indicated as * (*p* < 0.05).

As expected from the results for pattern, the SS1 scaffold configuration had an increased cell proliferation rate compared to SS3 (same design as 27), and significantly, to SS2. These results, shown in Figure 4, verified the Taguchi experimental design.

**Figure 5:**
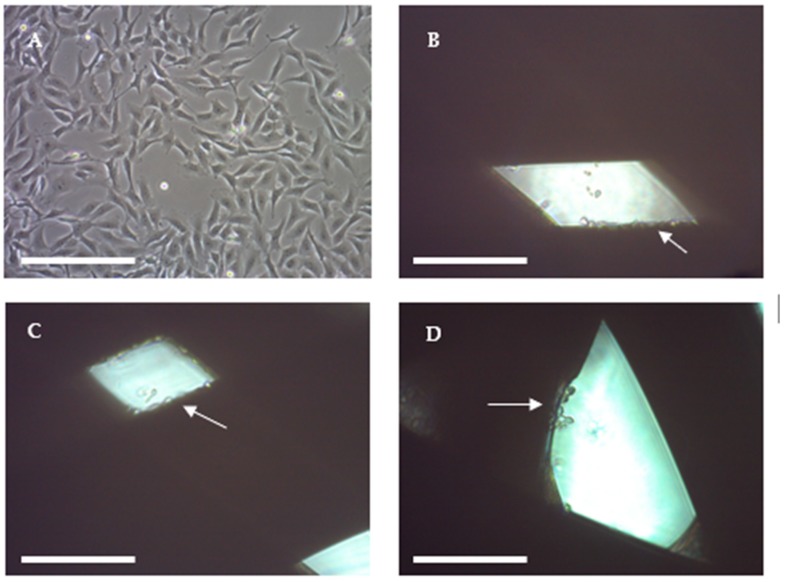
Optical microscope images of MDA-MB-231 cells attached to scaffold walls. (**A**) MDA-MB-231 cells on a 2D cell culture. (**B**) Cells attached to the SS1 configuration scaffold. (**C**) MDA-MB-231 cells attached to scaffold configuration 18. (**D**) Cells attached to scaffold configuration 25. White arrows indicate cells adhered to PLA filaments. Images from optical microscopy 100×. Scale bar: 0.25 mm.

The main objective here was to manufacture a scaffold that provided a high cell growth rate to further obtain BCSC enrichment. It is important to emphasize that, while a high proliferation rate does not mean a high proportion of BCSCs, the enrichment of this malignant subpopulation is required for their study. The more cells there are attached to the scaffold, the easier it will be to perform enrichment experiments. If a particular scaffold produces a good enrichment, a high absolute number of BCSCs will be collected. Consequently, those scaffold designs with an average cell proliferation rate ≥ 23% and a SE ≤ 2% were chosen. Following this criterion, configurations SS1, 18 and 25 were selected and are presented in Figure 5. Cells grown on 2D surfaces were extended in contrast with the 3D-cultured cells which appeared more rounded and smaller. 

### 2.3. Aldehyde Dehydrogenase Activity

Aldehyde dehydrogenases is a family of enzymes involved in aldehyde metabolism and the oxidation of exogenous and endogenous aldehydes into carboxylic acids [29]. Aldehyde dehydrogenase 1 (ALDH1) is considered to be an internal marker for stem and progenitor cells [12] and is used to isolate and identify CSCs [30]. ALDH1 is highly expressed in different types of cancer, including breast [31,32,33]. ALDEFLUOR^TM^ assay is a commercial test employed to identify and isolate CSCs of different types of cancers. 

MDA-MB-231 cells were cultured for 3 and 6 days on 2D adherent surfaces and on selected PLA scaffold configurations (SS1, 18, and 25). ALDEFLUOR^TM^ assay was performed to test if 3D cell culture increased ALDH+ cell population. 

**Figure 6:**
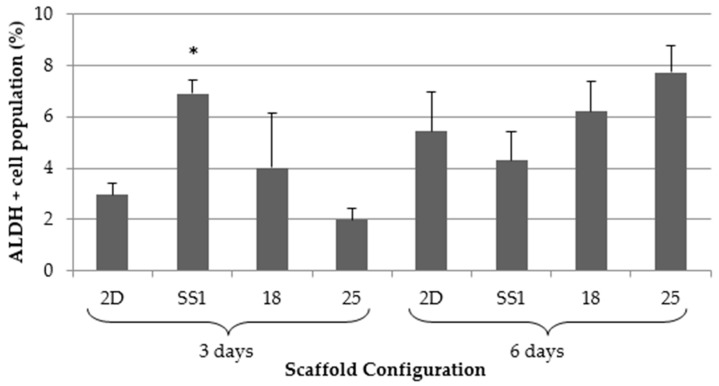
ALDEFLUO^TM^ assay results for three scaffold selected architectures and 2D control for 3 and 6 days. (*) Denotes significant (*p* < 0.05) differences between scaffold culture and 2D sample (*n* = 3).

As shown in Figure 6, the SS1 architecture significantly enriched the ALDH+ cell population compared to the 2D control after three days of culture. Scaffold configuration 18 exhibited a tendency to increase the ALDH+ population as compared to the 2D control, whereas design 25 presented a population reduction trend. In contrast, during six culture days, none of the architectures significantly increased the ALDH+ cell population compared to the monolayer cells. Designs 18 and 25 had a slight tendency to increase ALDH+ population, whereas SS1 decreased it. Therefore, these results show that the SS1 scaffold could be a useful tool to enrich ALDH+ populations in short culture timeframes. ALDH activity increase indicated an expansion in the BCSCs population, making the SS1 scaffold a good support for BCSCs study.

## 3. Discussion

BCSCs are a small population implicated in cancer recurrence, metastasis, and chemoresistance. New therapeutic approaches need to be found, but researchers have experienced difficulties in doing so because of their low representation in the tumor and traditional 2D cultures causes. Hence, 3D scaffolds appear to be a suitable alternative in which to culture and enrich this malignant subpopulation because they can mimic the physiological cell environment. 

PLA porous scaffolds were used to culture MDA-MB-231 triple negative breast cancer cells. Five parameters—layer height, infill density, infill pattern, infill direction, and flow—were analyzed to obtain a scaffold design that would be able to provide high cell proliferation. Twenty-seven different configurations were manufactured with a 3D printer following the Taguchi experimental design method. Some designs obtained smaller pore areas (less than 0.1 mm^2^) than other architectures with pore areas above 1 mm^2^. Besides this, some configurations had regular patterns with similar pore size, whereas others had non-regular patterns and irregular pores. Pore structure is an important characteristic to consider because it is directly related to cell growth and migration, nutrient flow, or vascularization [34]. 

TNBC cells were cultivated on 3D structures for three days and 2D cell culture was used as the control because the plastic dish is the main system used to cell culture in worldwide laboratories. All 3D configurations showed less cell proliferation than 2D cultures did. This is in line with previous studies [35,36]. Some investigations explained that cells can survive longer in 3D culture because of their slow growth, their migration through the pores and growth in the layers [36,37]. Ye et al. manufactured mineralized polyvinyl alcohol scaffolds and observed that MDA-MB-231 cells had higher proliferation in scaffolds with smaller pores [38]. Xiong et al. synthesized bacterial cellulose scaffolds and MDA-MB-231 cells showed a greater attachment, growth, proliferation, and spreading over those scaffolds with a diameter pore greater than 100 μm [34]. Our results have showed that the scaffold with the largest pores had a lower cell proliferation than the scaffolds with the smallest pores, which is in agreement with previous research [34,38]. Proliferation rate results were analyzed using Quantum XL software and the infill density and direction parameters that had a significant influence on cell proliferation were the optimal tested values of 70% and 45°, respectively. These results represented that proliferation was greater when cells had more available material and corners to attach to and grow on. It was described that scaffold geometry affects cell seeding and proliferation and must be optimized for each cell type [39]. Sobrat et al. demonstrated that orientation and layout of filament are significantly important for biological experiments. Moreover, they observed that cell seeding efficiency improves with stepped filament [40]. New designs with the optimal tested values were manufactured and the highest proliferation was obtained with the SS1 configuration, followed by configurations 18 and 25. These three architectures were selected to evaluate BCSC enrichment.

It was demonstrated that 3D printed PLA scaffold enhanced ALDH activity, which is used to identify a CSC niche. After three days of 3D culture, the SS1 configuration significantly increased the ALDH+ cell population when compared to the monolayer cells. These results indicated that scaffolds avoid differentiation of BCSC population. This could be the result of a cytoskeleton reorganization because of culture cells in a 3D system [22]. Interestingly, Rabiomet et al. and Feng et al. demonstrated that poly(ε-caprolactone) (PCL) electrospun scaffolds also increased ALDH+ niche, but that was after six days culturing with scaffolds [28,37]. Florczyk et al. manufactured chitosan and sodium alginate scaffolds and after 15 days of 3D culture, the structures promoted CSC-like cell growth [35]. Therefore, 3D printed PLA scaffolds can enrich BCSC niche in a MDA-MB-231 cell line in less time than other types of scaffolds can. Noreikaité et al. demonstrated that PLA electrospun scaffolds ensured a suitable environment for cell growth with mesenchymal stem cells which were capable of spreading after three days of cultivation [41].

In future investigations, it would be interesting to use PLA blends to mimic the extracellular matrix structure. PLA is very hydrophobic, but it can be modified with other polymers like collagen or hyaluronic acid [42,43,44]. PLA blends enhanced cell viability, endothelization, and cell morphology [45]. Archille et al. fabricated PCL electrospun scaffolds which incorporated and delivered a short RNA hairpin against the cell cycle specific protein Cdk2, decreasing their mRNA expression and cell proliferation [46]. Thus, PLA could be modified to improve the results obtained in the present study. 

In conclusion, FFF PLA scaffolds could be a useful tool with which to culture and enrich BCSCs. Consequently, 3D culture would allow BCSCs to be studied. 

## 4. Materials and Methods 

### 4.1. Scaffolds Design and Manufacture Process

SolidWorks^®^ (Dassault Systèmes SE, Suresnes, France) was the computer-aided design (CAD) software used to create a cylinder with 20 mm diameter and 2.4 mm height. Scaffold design was saved in a stereolithography (STL) file which was transferred to the computer-aided manufacturing (CAM) software BCN3D Cura (BCN3D Technologies©, Barcelona, Spain). Input process parameters were selected according the design of experiments.

### 4.2. Design of Experiment

Through Quantum XL (Digital Computations Inc., Orlando, FL, USA), Taguchi experimental design was chosen with the aim to analyze the effect of the input parameters, such as layer height, infill density, pattern, and direction and flow, on cell proliferation (Table 4). The parameter of infill density is the quantity of material founded in each layer, whereas the flow is the quantity of material that printer expelled. 

Taguchi is a statistical method, also known as a robust design method, developed by Genichi Taguchi. Taguchi helps to improve the quality of manufacturing processes. This statistical method is especially useful to reduce the number of experiments in processes with many input variables as 3D Printing based on FFF is.

The set parameters were printing temperature of initial layer (220 °C), printing temperature (200 °C), build plate temperature (30 °C), print speed (50 mm/s), retraction speed (40 mm/s), and nozzle diameter (0.3 mm). BCN3D Cura generated a G-code file of each scaffold configuration designed with the software and they were loaded in the 3D printer BCN3D Sigma Release 2017 (BCN3D Technologies©). PLA white (BCN3D Technologies©) was the biopolymer chosen to manufacture all scaffolds.

### 4.3. Material

Poly (lactic acid) (PLA, BCN3D Technologies©, Barcelona, Spain) was selected as the material for the experiments (Table 5). PLA is a biodegradable thermoplastic aliphatic polyester derived from renewable resources, such as corn starch or sugarcane, and has a melting point of about 173–178 °C with a glass transition of 60–65 °C. Degradation of PLA is produced by the hydrolysis of their ester linkages in physiological conditions. 

### 4.4. Cell Line

MDA-MB-231 triple negative breast cancer cells were obtained from the American Type Culture Collection (ATCC; Rockville, MD, USA). MDA-MB-231 cells were routinely grown in Dublecco’s Modified Eagle’s Medium (DMEM) supplemented with 10% fetal bovine serum (FBS) and 50 U/mL penicillin/streptomycin (Hyclone, Logan, UT, USA). Cells were kept at 37 °C and 5% CO_2_ atmosphere.

### 4.5. Three-Dimensional Cell Culture

Scaffolds were soaked overnight in 70% ethanol/water solution. The structures were washed two times with phosphate-buffered saline (PBS) (Hyclone) and exposed to UV light for 30 min. This sterilization process were followed based on previous work to avoid changes in the material properties [47]. 

Sterilized scaffolds were placed in non-adherent cell culture 12-well plates (Sartstedt, Nümbrecht, Germany) and immersed in a culture medium for 30 min at 37 °C and 5% CO_2_ atmosphere with the purpose to facilitate cell attachment. Then, the pertinent cell density was prepared in 50 μL of medium and the suspension was pipetted drop by drop over the scaffold centre. They were incubated for three hours at 37 °C and 5% CO_2_ atmosphere to allow cell attachment. After this incubation period, 1 mL of culture medium was added covering the PLA structures.

### 4.6. Scaffold Dimensional Characterization

Scaffolds were analyzed through digital images captured by Optical Microscope Nikon SMZ–745T attached to a digital camera CT3 ProgRes. The images were examined using the ImageJ® Software 1.5F (National Institutes of Health, Bethesda, MD, USA) and the pore area and the filament diameter were measured.

### 4.7. Cell Proliferation Assay

On adherent 12-well cell culture plates (Startstedt) and PLA scaffolds, 50,000 cells were seeded for 72 h. Scaffolds and adherent wells were washed two times with PBS and PLA structures were placed in new wells so as to ensure only attached cells would be tested. Then, 3-(4,5-dimethylthiazolyl-2)-2,5-diphenyltetrazolium bromide (MTT) assay was performed adding 1 mL DMEM and 100 μL MTT (Sigma-Aldrich, St. Louis, MO, USA) in each well for 150 min at 37 °C and 5% CO_2_ atmosphere. Only viable cells are capable of transform MTT into formazan crystals. After incubation, formazan crystals were dissolved with 1 mL dimethyl sulfoxide (Sigma-Aldrich) for 15 min under shaking. Three 100 μL aliquots from each well were pipetted into a 96-well plate and placed into a microplate reader (Bio-Rad, Hercules, CA, USA) where absorbance was measured at 570 nm.

### 4.8. Aldefluor Assay

On adherent 12-well cell culture plates (Startstedt) and PLA scaffolds, 50,000 cells were seeded for three days or 25,000 cells for six days. Scaffolds and adherent wells were washed two times with PBS and PLA structures were placed in new wells. Cells were detached using trypsin-EDTA (HyClone) at 37 °C and 5% CO_2_ atmosphere. A final concentration of 200,000 cells was needed to analyze the ALDH enzyme activity using the ALDEFLUOR^TM^ kit (Stem Cell Technologies, Durham, NC, USA). Following the manufacturer indications, cells were resuspended in ALDEFLUOR^TM^ assay buffer. There was a negative control for each sample to evaluate the background fluorescence using ALDH inhibitor ALDEFLUOR^TM^ diethylaminobenzaldehyde (DEAB). So, ALDEFLUOR^TM^ Reagent (BODIPY-aminoacetaldehyde; BAAA) was added to each cell suspension, and then, the half of each one was putted in other microcentrifuge tubes with DEAB, for their negative control. Samples were incubated at 37 °C for 45 min in the dark. Finally, all reagent was removed and incubated samples were resuspended in ALDEFLUOR^TM^ assay buffer. 

Cell Lab Quanta flow cytometer (Beckman Coulter Inc., Miami, FL, USA) was utilized to quantify the ALDH-positive cell population of all samples. The argon ion laser (488 nm) was used as a light source set at a power of 22 mW and the sheath rate was set at 4.17 µL/min. Green fluorescence was detected with fluorescent channel 1 (FL1) optical filter (dichroic/splitter, dichroic long-pass: 550 nm, band-pass filter: 525 nm, detection width 505 to 545 nm). Information of a minimum of 10,000 events was recorded in List-mode Data files (LMD) and analyzed using Flowing Software version 2.5.1 (Flowing Software, Turku, Finland). Data were not compensated.

First, side-scatter (SS) and electronic volume (EV) dot plots were executed and only single cells were selected, excluding aggregated and damaged cells (less than 5–10%). Then, SS and FL1 fluorescence dot plots from negative control samples were performed in order to determine background fluorescence. The ALDH-positive cells region was drafted at the rightmost plot zone and including only the 0.5% of total single cell population. BAAA samples were equally processed and ALDH-positive cells region of respective controls were adopted to identify the percentage of population with high ALDH activity for each sample. 

### 4.9. Statistical Analysis

All results were confirmed by at least three independent experiments. Data are expressed as mean ± standard error (SE). Data were analyzed by Student *t* test. P value is shown in results when significance was reached (*p* < 0.05). Ten copies (*n* = 10) of each DOE configuration were printed to perform characterization and cell proliferation assays.

## 5. Conclusions

The results of this work indicate that MDA-MB-231 cells can be cultured on PLA 3D printed scaffolds and support PLA as being a suitable material for 3D cell culture. PLA scaffolds with smaller pores produced higher proliferation, proving to be good physical supports for TNBC cells. Moreover, these structures provide for the expansion of BCSC niche. In conclusion, PLA scaffolds may be useful to culture BCSCs, thus making their growth and cultivation possible. This can facilitate research into this malignant subpopulation.

## Figures and Tables

**Table 1:** Design and process parameters of PLA scaffold configurations resulting from Taguchi experimental design.

Configuration	Layer Height (mm)	Infill Density (%)	Infill Pattern	Infill Direction (°)	Flow (%)
**1**	0.10	50	Zigzag	45	80
**2**	0.10	50	Zigzag	45	90
**3**	0.10	50	Zigzag	45	100
**4**	0.10	60	Grid	60	80
**5**	0.10	60	Grid	60	90
**6**	0.10	60	Grid	60	100
**7**	0.10	70	Triangles	90	80
**8**	0.10	70	Triangles	90	90
**9**	0.10	70	Triangles	90	100
**10**	0.15	50	Grid	90	80
**11**	0.15	50	Grid	90	90
**12**	0.15	50	Grid	90	100
**13**	0.15	60	Triangles	45	80
**14**	0.15	60	Triangles	45	90
**15**	0.15	60	Triangles	45	100
**16**	0.15	70	Zigzag	60	80
**17**	0.15	70	Zigzag	60	90
**18**	0.15	70	Zigzag	60	100
**19**	0.20	50	Triangles	60	80
**20**	0.20	50	Triangles	60	90
**21**	0.20	50	Triangles	60	100
**22**	0.20	60	Zigzag	90	80
**23**	0.20	60	Zigzag	90	90
**24**	0.20	60	Zigzag	90	100
**25**	0.20	70	Grid	45	80
**26**	0.20	70	Grid	45	90
**27**	0.20	70	Grid	45	100

**Table 2:** Pore area (mm^2^) and filament diameter (mm) values of each PLA scaffold configuration obtained from scaffold image analyses.

Configuration	Pore Area (mm^2^)	Filament Diameter (mm)
**1**	0.353 ± 0.010	0.243 ± 0.006
**2**	0.337 ± 0.009	0.301 ± 0.009
**3**	0.301 ± 0.006	0.331 ± 0.007
**4**	0.402 ± 0.025 (Irregular Pores; From 0.055 to 0.753)	0.287 ± 0.007
**5**	0.384 ± 0.032 (Irregular Pores; From 0.065 to 0.794)	0.294 ± 0.010
**6**	0.361 ± 0.031 (Irregular Pores; From 0.047 to 0.748)	0.338 ± 0.012
**7**	0.263 ± 0.021 (Irregular Pores; From 0.029 to 0.550)	0.348 ± 0.011
**8**	0.240 ± 0.017 (Irregular Pores; From 0.081 to 0.505)	0.359 ± 0.010
**9**	0.197 ± 0.018 (Irregular Pores; From 0.044 to 0.546)	0.367 ± 0.007
**10**	1.376 ± 0.013	0.298 ± 0.008
**11**	1.322 ± 0.009	0.341 ± 0.007
**12**	1.216 ± 0.010	0.378 ± 0.008
**13**	0.365 ± 0.034 (Irregular Pores; From 0.065 to 0.825)	0.334 ± 0.013
**14**	0.335 ± 0.031 (Irregular Pores; From 0.054 to 0.786)	0.354 ± 0.015
**15**	0.296 ± 0.032 (Irregular Pores; From 0.054 to 0.715)	0.370 ± 0.011
**16**	0.074 ± 0.002	0.332 ± 0.007
**17**	0.069 ± 0.002	0.348 ± 0.007
**18**	0.041 ± 0.002	0.408 ± 0.007
**19**	1.736 ± 0.029	0.329 ± 0.007
**20**	1.714 ± 0.027	0.340 ± 0.004
**21**	1.611 ± 0.025	0.374 ± 0.008
**22**	0.125 ± 0.003	0.295 ± 0.005
**23**	0.098 ± 0.002	0.312 ± 0.005
**24**	0.090 ± 0.002	0.338 ± 0.005
**25**	0.202 ± 0.019 (Irregular Pores; From 0.039 to 0.437)	0.364 ± 0.011
**26**	0.187 ± 0.018 (Irregular Pores; From 0.029 to 0.390)	0.373 ± 0.014
**27**	0.180 ± 0.016 (Irregular Pores; From 0.046 to 0.381)	0.394 ± 0.014

**Table 3:** Scaffold configurations resulting from the Taguchi experimental design analysis. (Scale bar: 2 mm).

Configuration	Selected Values	Pore Area (mm^2^)	Filament Diameter (mm)	Microscopic Image
**SS1**	Layer Height: 0.2 mm Infill Density: 70% Infill Pattern: Zigzag Infill Direction: 45° Flow: 100%	0.054 ± 0.002	0.483 ± 0.009	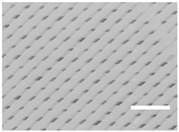
**SS2**	Layer Height: 0.2 mm Infill Density: 70% Infill Pattern: Triangles Infill Direction: 45° Flow: 100%	0.224 ± 0.020 (Irregular pores; From 0.041 to 0.491)	0.387 ± 0.010	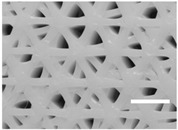

**Table 4:** Tested values of each analyzed parameter to obtain the best cellular growth rate.

Parameter	Tested Values
**Layer Height**	0.1, 0.15 and 0.2 mm
**Infill Density**	50, 60 and 70%
**Infill Pattern**	Grid, Triangles and Zigzag
**Infill Direction**	45, 60 and 90°
**Flow**	80, 90 and 100%

**Table 5:** Poly (lactic acid) properties.

Material(#)	Molecular Weight (g/mol)	Young’s Modulus (MPa)	Strain at Break (%)	Degradation Time (Months)
**PLA**	30,000	108	3.5	≈12

## Abbreviations

2D	Two-dimensional
3D	Three-dimensional
ALDH	Aldehyde Dehydrogenase
AM	Additive Manufacturing
BCSC	Breast cancer stem cell
CAD	Computer-aided design
CAM	Computer-aided manufacturing
CSC	Cancer stem cell
DMEM	Dulbecco’s modified eagle’s medium
ECM	Extracellular matrix
FBS	Fetal bovine serum
FFF	Fused filament fabrication
MTT	3-(4,5-dimethylthiazolyl-2)-2,5-diphenyltetrazolium bromide
PBS	Phosphate-buffered saline
PCL	Poly(ε-caprolactone)
PLA	Poly (lactic acid)
SS	Selected scaffold
STL	Stereolithography
TNBC	Triple negative breast cancer
